# Utilizing the nanosecond pulse technique to improve antigen intracellular delivery and presentation to treat tongue squamous cell carcinoma

**DOI:** 10.4317/medoral.22227

**Published:** 2018-04-24

**Authors:** Cen Gao, Xingxing Zhang, Jian Chen, Jiayuan Zhao, Yanmei Liu, Jue Zhang, Jing Wang

**Affiliations:** 1 Department of Periodontology, School of Stomatology, Lanzhou University, Lanzhou, Gansu, China; 2 Department of Pediatric Surgery, The First Hospital of Lanzhou University, Lanzhou, Gansu, China; 3 Academy for Advanced Interdisciplinary Studies, Peking University, Beijing, China

## Abstract

**Background:**

Tongue squamous cell carcinoma is the most common squamous cell carcinoma of the head and neck. Immunotherapy has great potential in the treatment of tongue squamous cell carcinoma because of its unique advantages. However, the efficacy of immunotherapy is limited by the efficiency of antigen phagocytosis by immune cells.

**Material and Methods:**

We extracted dendritic cells (DCs) from human peripheral blood. Utilizing a nanosecond pulsed electric field (nsPEF), we deliver the tumour lysate protein into DCs and then incubate the DCs with PBMCs to obtain specific T cells to kill tumour cells. The biosafety of nsPEF was evaluated by the ANNEXIN V-FITC/PI kit. The efficacy of lysate protein delivery was evaluated by flow cytometry. The antitumour efficacy was tested by CCK-8 assay.

**Results:**

The nsPEF of the appropriate field strength can significantly improve the phagocytic ability of DCs to tumour lysing proteins and have good biosafety. The tumour cell killing rate of the nsPEF group was higher than the other group (*p*< 0.05).

**Conclusions:**

Utilizing nsPEF to improve the phagocytic and presenting ability of DCs could greatly activate the adaptive immune cells to enhance the immunotherapeutic effect on tongue squamous cell carcinoma.

** Key words:**Dendritic cell, nsPEF, immunotherapy, squamous cell carcinoma.

## Introduction

Cancer is one of the deadliest human diseases and has proved difficult to treat. According to WHO statistics, the worldwide morbidity and mortality rates of cancer are high (1). Traditional treatments, such as surgery, radiotherapy and chemotherapy, have only increased the median progression-free survival to some extent; however, they are still unable to overcome drug resistance, metastasis and serious side effects and defects (2-5). In recent years, tumour immunotherapy has attracted increasing interest due to its high efficiency, high specificity and cascade amplification. The key to tumour immunotherapy is the modification of primary presenting cells (6-11). Antigen presenting cells (APCs), which are a subtype of immune cells, include dendritic cells (DCs), macrophages, and B cells. APCs capture exogenous or endogenous proteins and in turn activate the adaptive immune cells to generate an inflammatory response or tolerance immune response (12). In tumour immunotherapy, APCs digest tumour proteins and present them to immature T cells to activate their tumour killing function (13,14). Immunotherapy based on DCs has shown excellent treatment efficacy for many cancers, such as gliomas, cervical cancer, prostate cancer, and malignant melanoma (15-17). However, the weak immunogenicity of tumour antigens and the low efficiency of antigen phagocytosis potently limit the effect of immunotherapy. Many research groups have attempted to overcome these problems (12). For example, nanotubes have been used to carry tumour lysate proteins to enhance the phagocytosis of tumour lysate proteins by DCs, thereby increasing the anti-tumour effect (18). HADJATI *et al.* strengthened immunotherapy by using mouse toxoplasmosis to enhance the antigen phagocytosis and presentation function of DCs. LANGER *et al.* (19) used microfluidic technology to induce transient cell membrane perforation and increase antigen uptake, thereby improving the efficacy of tumour immunotherapy. JAMAL *et al.* (20) fabricated a vehicle based on carbon nanotubes for delivering tumour antigens and adjuvants to enhance the effectiveness of tumour immunotherapy (21).

Nanosecond pulsed electric field (nsPEF) can generate instantaneous cell membrane perforation to efficiently mediate macromolecule entry into cells (22,23). For example, nsPEF can be used to mediate plasmid entry into cells and support efficient expression. The synergistic effects of nsPEF combined with gemcitabine have been shown to promote the treatment of malignant melanoma and oral squamous cell carcinoma (21-24). In addition, this modality has many advantages, such as minimal side effects, high biocompatibility and tumour-specific cytotoxicity (25). Because of these unique advantages, we established a method based on nsPEF technology to improve the treatment of tongue squamous cell carcinoma. Furthermore, we confirmed the improvement in the antigen presenting ability of DC phagocytes by nanosecond pulsed stimulation.

## Material and Methods

- Mononuclear cells (PBMC) were extracted from the peripheral blood of participants

Venous blood (30 mL) was extracted from healthy volunteers using a disposable anticoagulation tube (BD, Lake Franklin, New Jersey, USA) under the approval of the Institutional Review Board (IRB) at the Hospital of Stomatology of Lanzhou University, Lanzhou, Gansu, China. Volunteers consented to the use of their blood samples for this study before collection. Under sterile conditions, the anticoagulation tube was removed and placed in a 50 mL centrifuge tube (Corning, Corning, New York, USA). The peripheral blood mononuclear cells (PBMCs) were extracted by Human Lymphocyte Separation Medium (TBD, Tianjin, China). The cells were suspended in RPMI 1640 (Gibco, Grand Island, New York, USA) + 10% FBS (Gibco, Grand Island, New York, USA) complete medium, and the final concentration of the PBMCs was 2 × 105/mL.

- Isolation and induction of mature DCs

Freshly isolated PBMCs were resuspended in RPMI complete medium, and the cell density was adjusted to 4 × 106/mL (5 mL). Then, the cells were cultured in a T25 flask (Corning, Corning, New York, USA) (37 °C, 4 h, 5% CO2) in an incubator, and the culture supernatant and non-adherent cells were discarded by sterile straw aspiration. The bottle was carefully washed with preheated RPMI 1640 + 10% FBS complete medium to obtain relatively pure adherent mononuclear cells, and 5 mL of RPMI 1640 + 10% FBS complete medium was added to the culture flask. Then, 5 μL of rhGM-CSF (R&D Systems, Minneapolis, Minnesota, USA) and 4 μL of rhIL-4 (Beyotime Biotechnology, Shanghai, China) were added. After culturing in the incubator for 6 d (37 °C, 5% CO2), the immature DCs were obtained.

TNF-α (Beyotime Biotechnology, Shanghai, China) (20 ng/mL) was added to the immature DCs, and after 2 d of culture, the DCs became larger and clustered into mature DCs.

- Preparation of Cal-27 lysate protein 

Cal-27 tongue squamous cell carcinoma cells (ATCC) were maintained in Dulbecco’s modified Eagle medium (DMEM) (Gibco, Grand Island, New York, USA) supplemented with 10% FBS, L-glutamine (Beyotime Biotechnology, Shanghai, China) and penicillin/streptomycin (Beyotime Biotechnology, Shanghai, China) at 37 °C, 5% CO2 and saturated humidity. The cells were cultured to the logarithmic phase and passaged and cryopreserved by routine methods.

To digest the Cal-27 cells in the logarithmic growth phase, the cells were treated with 0.25% trypsin (HyClone, Logan, Utah, USA). Then, the culture medium was removed, the sample was washed with D-Hank’s buffer solution 2 times and centrifuged (1000 r/min, 5 min), and the cell sedimentation was then collected. Cell lysis solution (Beyotime Biotechnology, Shanghai, China) was then added to each well at a volume of 1 mL to obtain the lysate. The cell mixture was placed at 4 °C for 0.5 h and then centrifuged for 5 min at 12,000 g. The supernatant was Cal-27 cell lysate protein, and 50 μL was removed and placed in the tube to determine the protein concentration. The remainder was stored at -80 °C. The protein concentration was determined using the BCA kit (Beyotime Biotechnology, Shanghai, China).

- Tumour antigen-sensitized DCs

Immature DCs were harvested for 6 d, and the cells were counted. The cell concentration was adjusted to 2 × 105/mL with 10% FBS containing RPMI 1640 medium and added to 24-well plates (Corning, Corning, New York, USA) at a volume of l mL per well. A total of 4 wells were used as follows: DC well, TumourP-treated well, nsPEF-treated well, TumourP and nsPEF-treated well. The DC well had no stimulant, and the other three wells were treated with TumourP, nsPEF stimulation, TumourP and nsPEF stimulation. The protein concentration in each group was 0.1 μg/mL to sensitize the DCs. In addition, 20 ng/mL TNF-α was added to obtain mature DCs at 37 °C under the 5% CO2 culture conditions for 2 d.

- Homologous mixed lymphocyte reactions

Peripheral blood samples were collected from the same source as the DCs of the healthy volunteers. PBMCs were separated using the Ficoll density gradient method, and the cell density was adjusted to 2 × 106/mL. Different antigens and mature DCs (DC: PBMC=1:10) were added and incubated for 3-5 d (D 37 °C, 5% CO2) to obtain mixed lymphocytes.

- Detection of the killing effect of mixed lymphocytes on Cal-27 cells

Cal-27 cells were incubated in 96-well plates (Corning, Corning, New York, USA), with 5000 cells per well. After incubation for 4 h (37 °C, 5% CO2), adherent cells were regarded as target cells (T). After co-culture with the DCs, the PBMCs were extracted as effector cells (E). The cell density was adjusted to 2 × 106/mL with RPMI 1640 complete medium, and we then added PBMCs to each well in different proportions (E: T= 40: 1, 20: 1, 10: 1, 5: 1). Each group consisted of 6 parallel wells. After incubation for 2-3 d (37 °C, 5% CO2), the suspended lymphocytes were removed from each well. Almost no lymphocytes were observed in each well under a microscope.

**Figure 1:**
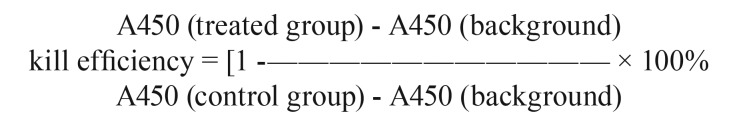
Formula

- Tumour killing rate detected by CCK-8:

A total of 100 μL of RPMI complete medium was added to a 96-well plate with a blank well containing only medium and CCK-8 reagent and no targets as the background value (DOJINDO, Kyushu, Japan). Then, 10 μL of CCK-8 solution was added to each well and reacted without light for 4 h at 37 °C. The A450 absorbance value was then measured. The kill rate was calculated using the following formula: (Fig. 1).

- Flow cytometry to detect DC phagocytosis of the tumour lysate protein

Following the instructions of the ReadLink antibody labelling kits (AAT Bioquest, Sunnyvale, CA, USA), Cal-27 lysate protein (1 mg/mL) was mixed with FITC dye molecules and dissolved in reaction buffer. Exposure to light was avoided for 30-60 min. FITC-labelled tumour protein (TumourP-FITC) was obtained by adding an appropriate quencher for 10 min to remove unbound FITC.

We harvested immature DCs after 6 d. The cells were placed in a 24-well plate at a density of 3-5 × 105 cells/per well. The wells were grouped as follows: 1) control DCs, which was without antigen; 2) adsorptive fluorescence control group, in which TumourP-FITC with a protein concentration of 1 μg/mL was added to the DCs in an ice bath for 4 h; 3) phagocytic group, in which DCs were added to a protein concentration of 1 μg/mL FITC and incubated at 37 °C under 5% CO2 for 4 h; and 4) a nanosecond pulsed group, in which TumourP-FITC with a protein concentration of 1 μg/mL was added and incubated for 4 h at 37 °C under 5% CO2 for protein phagocytosis. After 4 h, the cells were collected and centrifuged at 1500 r/min for 10 min. The supernatant was removed and the cells were collected and washed with D-Hank’s buffer twice to remove the non-phagocytic protein. The positive rate and mean fluorescence intensity of DC phagocytosis with FITC-TumourP were then detected by flow cytometry.

- nsPEF acts on the safety of immature DCs

DC apoptosis was detected using the ANNEXIN V-FITC/PI kit (Solarbio Life Sciences, Beijing, China). The mature DCs were resuspended in pre-cooled 1 × PBS (HyClone, Logan, Utah, USA) (4 °C) and centrifuged at 2000 rpm for 5 to 10 min to wash the cells. A total of 300 μL of 1 × Binding Buffer suspension cells was added. After adding 5 μL of Annexin V-FITC, the cells were incubated for 5 min, and 5 μL of PI staining was then added for 5 min before detection. DC apoptosis was detected by flow cytometry.

- Statistical analysis

More than three independent experiments are conducted for each experiment. All data were analysed by SPSS 16.0 through t test method. *P* < 0.05 was considered statistically significant.

## Results

- Extraction of dendritic cells and acquisition of tumour lysate proteins

**Figure 2:**
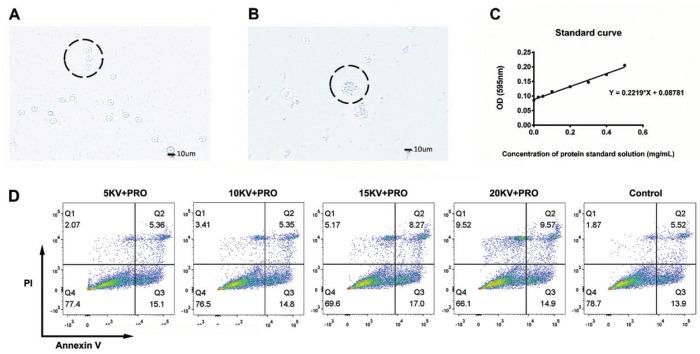
Extraction of lysate proteins from Cal-27 cells and the biosafety of nsPEF. (A) Mononuclear cells extracted from peripheral blood. (B) Dendritic cells were obtained by *in vitro* culture and cytokine stimulation. (C) The concentration of Cal-27 lysate protein was measured by the BCA Protein Assay Kit. The standard curve is shown in the figure. (D) nsPEF affects the survival of immature DCs (n≥3 independent experiments).

We collected blood samples from the volunteers and extracted PBMCs using a human peripheral blood lymphocyte separation solution. As shown in Fig. 2A, mononuclear cells were obtained by adherent separation and observed via optical microscopy. Dendritic cells were obtained by the induction of rhGM-CSF and rhIL-4 cytokines. We observed the cell characteristics via microscopy. As shown in Fig. 2B, the results indicated that the number and volume of cells increased. The dendritic protrusion appeared on the cell surface, and cell aggregation was observed. The results of the above cell morphological studies indicated that DCs were successfully extracted and induced. The experiment was repeated three times.

The BCA results are presented in Fig. 2C, and they show that an appropriate concentration of Cal-27 tumour lysing protein was obtained to meet the needs of the follow-up analyses. The experiment was repeated three times.

- nsPEF affects the survival of immature DCs

The cell apoptotic assay results are shown in Fig. 2D. These data suggest that in the range of 5-10 kV, the ratio of Annexin V to PI positive cells did not change significantly between the experimental group and the control group. The results revealed that the pulse generated a reversible instantaneous hole on the cell membrane and did not cause cell apoptosis. In the 15-20 kV range, the ratio of Annexin V to PI positive cells increased slightly, indicating that the nsPEF in this range caused a small amount of irreversible membrane perforation and cell apoptosis. The experiment was repeated three times.

- Flow detection of DC phagocytosis of tumour lysate proteins

**Figure 3:**
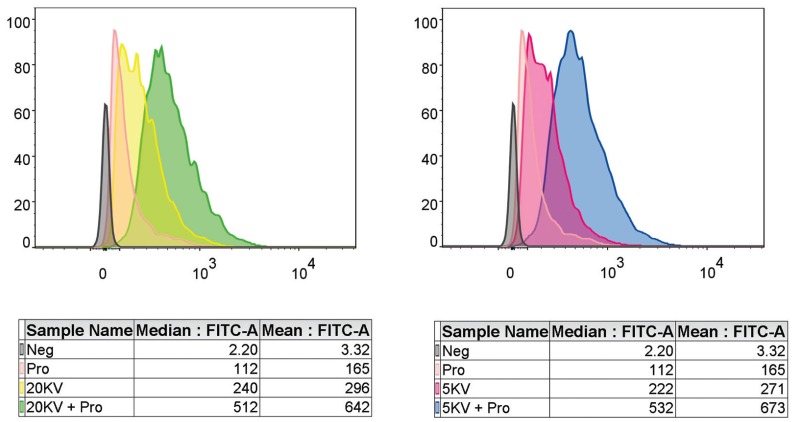
Flow detection of DC phagocytosis of tumour lysate proteins. (n≥3 independent experiments).

The flow detection experimental results (Fig. 3) show that the phagocytic volume of tumour lysing protein is increased by one fold compared with that of the non-nsPEF group with 5 kV and 20 kV nanosecond pulsed fields. These results suggest that a nsPEF of the appropriate field strength can significantly improve the phagocytic ability of DCs to tumour lysing proteins. The experiment was repeated three times.

- Effect of mixed lymphocytes on tumour cells

**Figure 4:**
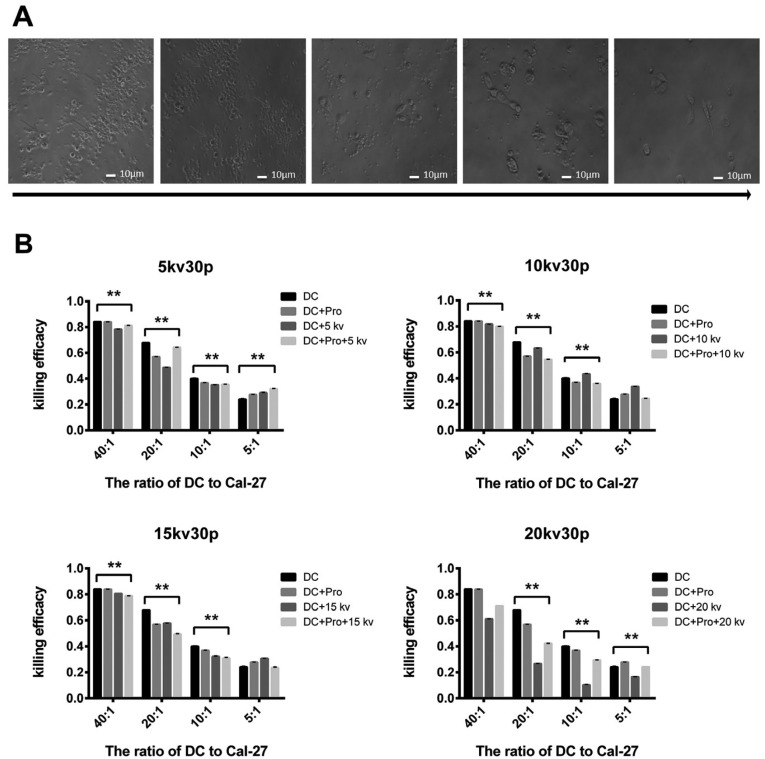
Effect of mixed lymphocytes on tumour cells. (A) Mature DCs sensitized PBMCs to obtain mixed lymphocytes containing specific T cells, which were co-cultured with Cal-27 cells, and the results of light microscopy are shown in the figure. From left to right, the ratio of lymphocytes to Cal-27 cells decreases. The red arrow points to cancer cell death fragments. (B) Killing efficacy (*p*<0.05). All data are presented as means± standard deviations (n≥3 independent experiments).

As shown in Fig. 4A, Cal-27 cells were observed to adhere to specific T cells, died after a period of time, and were broken down into cell debris. The results of the cytotoxicity test are presented in Fig. 4B. The tumour cell killing rate of the experimental group was increased by the application of nsPEF. The experiment was repeated three times.

## Discussion

Previous studies have shown that the weak immunogenicity of tumour antigens and the low efficiency of antigen phagocytosis potently limit the effect of immunotherapy. We utilized nsPEF, which assists macromolecule entry into the cell, to increase the efficiency of antigen phagocytosis and improve immunotherapy.

Loaded tumour proteins are the basis of tumour immunotherapy, and different antigen loading methods will affect the major histocompatibility complex type I and type II antigen presentation pathways, respectively, which triggers CD8+ and CD4+ T lymphocyte activation. At present, the loading method primarily includes protein polypeptide delivery, necrotic tumour cells, tumour lysates, and genetic engineering. Protein polypeptide delivery and genetic engineering have specific advantages, but resolving the tumour heterogeneity of the immune escape is difficult because of the single phenotype. Therefore, considering the heterogeneity of the tumour and the specificity of the immune cell phenotype and other factors, we chose to load tumour lysing proteins, which include all the tumour antigen phenotypes (26). We used tumour lysates and protease inhibitors to extract tumour lysate proteins. This method can be used to obtain all the tumour epitopes of tumour cells and is suitable for clinical application.

The cell membrane is an important barrier to maintain cell viability and function. Its main structure consists of a phospholipid bilayer layer that has a degree of mobility. The cell membrane generates transient holes under appropriate external stimulation. However, if the external stimulation is too strong, it will cause irreversible damage to the cell membrane structure, which will lead to cell apoptosis and necrosis. According to previous studies, the nsPEF can enhance the efficiency of extracellular macromolecule cell entry under appropriate parameters by generating transient holes in the cell membrane. To obtain the biological completeness range of the nsPEF intensity, we used an apoptotic kit to perform flow cytometry on the cells after treatment. The results demonstrated that our experimental protocol has good biosecurity.

After confirming the biosecurity of nsPEF, we evaluated the phagocytosis of tumour lysing protein, which is the key step in the antigen presentation of DCs. After phagocytosis, the DCs can treat the antigen and then present to the lymphocytes through the main histocompatibility antigen presentation pathway, thus causing subsequent antigen-specific immune responses. The phagocytosis process of the DCs is regulated by the identification of the antigen, which is affected by the different phenotypes or physical and chemical properties. The nsPEF causes instantaneous perforation of the membrane, and biological macromolecules, including various antigens, can enter the cytoplasm through free diffusion. To evaluate this effect, we used flow cytometry for detection. We used a method reported in the literature to obtain fluorescent labelled Cal-27 lysate protein in advance, apply nanosecond pulses at preset parameters, and perform flow cytometry. The average fluorescence intensity of the cells is positively correlated with cell phagocytosis. The results suggest that a nsPEF of the appropriate field strength can significantly improve the phagocytic ability of DCs for tumour lysing proteins.

T lymphocyte-mediated target cell killing is the ultimate step in tumour immunotherapy and is the most fundamental indicator of antitumour efficacy. The results of the cytotoxicity test showed that the tumour cell killing rate of the experimental group was increased by the application of nsPEF. This killing process is based on the phagocytosis and presentation of DC antigens; thus, the initial T cells in combination with DCs presented the antigen, which resulted in activation, followed by proliferation and differentiation into cytotoxic T cells under the action of cytokines. The final cytotoxic T-cell-secreting adhesion molecules bind to the corresponding target cells through the perforin/granzyme pathway and Fas/FasL pathway to kill the target cells.

The phenotypic differences between tumour cells and normal cells are obscure, and killing tumour cells without side effects is difficult. In addition, the proliferation and progression of tumour cells are uncontrollable; therefore, completely eliminating the tumour with surgical treatment, radiotherapy, chemotherapy and other traditional methods is difficult. The immune system has a specific antitumour ability. The immune response can be amplified through a cascade and has the characteristics of immune memory; therefore, tumour immunotherapy has the potential to completely cure tumours. However, a number of challenges remain for immunotherapy due to the adaptation of tumour tissue to the patient’s own immune system, including the low immunogenicity of tumour antigens and low efficiency of antigen presentation. To overcome these problems, we utilized nsPEF technology to improve tumour immunotherapy. The results confirmed that the application of nsPEF can promote the phagocytosis of tumour proteins and improve the immunogenicity of tumour antigens, thereby enhancing the antitumour effect of lymphocytes. In addition, the nsPEF technique has good biosafety and is noninvasive. In summary, this modality and the related mechanisms may be valuable for the future development of immunotherapies and can provide insights for tumour treatment.
